# Current Progresses and Challenges of Immunotherapy in Triple-Negative Breast Cancer

**DOI:** 10.3390/cancers12123529

**Published:** 2020-11-26

**Authors:** Karan Mediratta, Sara El-Sahli, Vanessa D’Costa, Lisheng Wang

**Affiliations:** 1 Department of Biochemistry, Microbiology and Immunology, Faculty of Medicine, University of Ottawa, 451 Smyth Road, Ottawa, ON K1H 8M5, Canada; kmedi072@uottawa.ca (K.M.); selsa056@uottawa.ca (S.E.-S.); 2 Centre for Infection, Immunity and Inflammation, University of Ottawa, 451 Smyth Road, Ottawa, ON K1H 8M5, Canada; 3 Ottawa Institute of Systems Biology, University of Ottawa, 451 Smyth Road, Ottawa, ON K1H 8M5, Canada

**Keywords:** triple-negative breast cancer, immunotherapy, immune checkpoint inhibitor, combination therapy, tumor microenvironment, cancer nanomedicine, tumor antigens, chimeric antigen receptor, cancer metabolism

## Abstract

**Simple Summary:**

The breakthrough of immunotherapy in melanoma has generated a glimmer of hope for lethal triple negative breast cancer (TNBC). This review summarizes the recent advances, challenges and potential new approaches of immunotherapy in TNBC.

**Abstract:**

With improved understanding of the immunogenicity of triple-negative breast cancer (TNBC), immunotherapy has emerged as a promising candidate to treat this lethal disease owing to the lack of specific targets and effective treatments. While immune checkpoint inhibition (ICI) has been effectively used in immunotherapy for several types of solid tumor, monotherapies targeting programmed death 1 (PD-1), its ligand PD-L1, or cytotoxic T lymphocyte-associated protein 4 (CTLA-4) have shown little efficacy for TNBC patients. Over the past few years, various therapeutic candidates have been reviewed, attempting to improve ICI efficacy on TNBC through combinatorial treatment. In this review, we describe the clinical limitations of ICI and illustrate candidates from an immunological, pharmacological, and metabolic perspective that may potentiate therapy to improve the outcomes of TNBC patients.

## 1. Introduction

Breast cancer alone accounts for 30% of all female cancers and remains one of the leading causes of cancer-related deaths globally, with 626,679 deaths in 2018 alone [1,2]. Triple-negative breast cancer (TNBC) is the most refractory subtype, which accounts for 11.2% of new breast cancer cases, but disproportionately accounts for the majority of breast cancer-related deaths [3]. Chemotherapy remains the current mainstay treatment due to the lack of specific targets for TNBC. However, it is often associated with short-lived clinical responses [4], systemic toxicity [5] and enrichment of cancer stem cell (CSC) populations. CSCs are capable of self-renewal, differentiation, metastasis, and regeneration of a new tumor [6]. Moreover, CSCs possess an abundance of drug resistance mechanisms, including the expression of several ATP-binding cassette (ABC) transporters that contribute to the poor clinical outcomes associated with TNBC [7,8].

It is evident that effective treatment of TNBC would depend upon the elimination of CSC populations. In recent years, the emergence of immunotherapy (IT) has offered new perspectives in the treatment and management of TNBC. Despite the lack of targeted therapies for TNBC, recent studies suggest that TNBC is the most immunogenic breast cancer subtype [9,10,11]. In one study, TNBC was reported to have a higher expression of Programmed Death-Ligand 1 (PD-L1), an immune checkpoint molecule that contributes to immune evasion [12]. Through immunohistochemistry staining, TNBC tumors have been shown to exhibit higher numbers of intratumoral and stromal tumor-infiltrating lymphocytes (TILs) [13]. PD-L1 expression has been correlated with high levels of TILs, an association that has been considered as a favorable indicator for TNBC patients’ prognosis [14,15]. Evaluating PD-L1 expression using immunohistochemistry assays such as VENTANA SP142 or Dako 22C3 has made it possible to identify patients who may benefit from immune checkpoint inhibition [16]. Other immune modulating receptors have also been identified as attractive targets for PD-L1^−^ and/or TIL^−^ tumors. Hallmarks of TNBC include dysregulated tumor vasculature, genomic instability, aberrant cell signaling, and deregulation of cellular energetics, each of which could be potential pharmacological targets for combination with immunotherapy. In this review, we discuss the progresses and challenges associated with the present modalities of immunotherapy with respect to TNBC and CSCs.

## 2. Immune Checkpoint Inhibition: Therapeutic Strategies

### 2.1. PD-1/PD-L1 Axis

**Figure 1:**
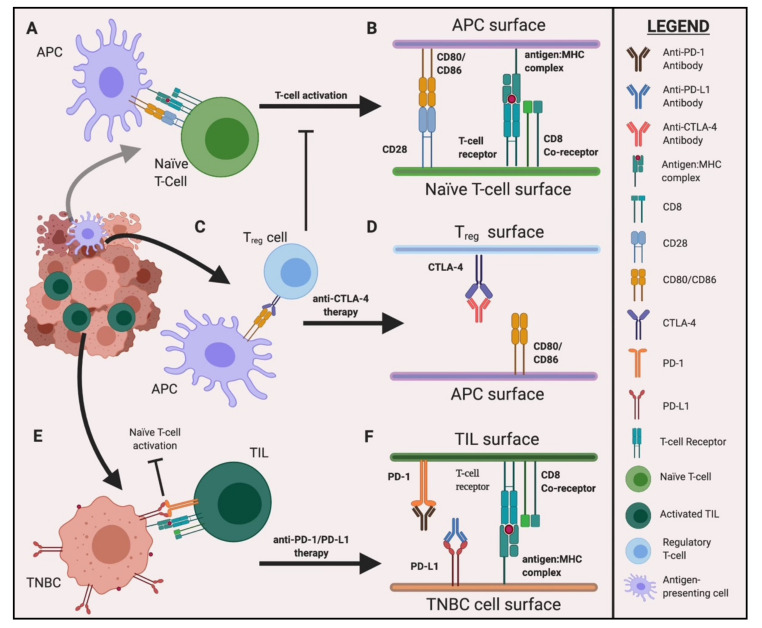
Possible mechanism of immune checkpoint inhibitors in triple-negative breast cancer (TNBC). Tumors consist of heterogenous cell populations including different types of immune infiltrating cells that undergo proliferation and apoptosis. (**A**) Tumor-infiltrating antigen-presenting cells (APCs) display tumor-associated antigens (TAAs) on their major histocompatibility complexes (MHC), which activate antigen-specific naïve T-cells. (**B**) Cluster of differentiation 80 (CD80) on the APC and cluster of differentiation 28 (CD28) are both co-signaling molecules necessary for T-cell activation and expansion. (**C**) CD80 molecules on tumor-infiltrating APCs preferentially bind to cytotoxic T lymphocyte protein-4 (CTLA-4), constitutively expressed on regulatory T cells (T_reg_) that commonly recruit myeloid-derived suppressor cells (MDSCs) to the tumor microenvironment (TME) to inhibit T-cell activation. (**D**) Anti-CTLA-4 antibodies inhibit T_reg_ activation and enhance anti-tumor activity. (**E**) Tumor cells express programmed death ligand-1 (PD-L1) and tumor-infiltrating lymphocytes express its receptor, PD-1. The interaction between PD-1 and PD-L1 inactivates T-cell activation/expansion within the TME. (**F**) Anti-PD-1 or anti-PD-L1 antibodies prevent PD-1/PD-L1 engagement, thereby inhibiting the suppressive signals and promoting anti-tumor immunity. Figure created with BioRender.com.

Immune checkpoint inhibitors (ICIs) have been considered as viable candidates for the treatment of TNBC. Effector T-cells express the Programmed Death 1 (PD-1) cell surface receptor, which interacts with its ligand, PD-L1 (Figure 1). PD-L1 is normally expressed on the surfaces of dendritic cells and macrophages, and binding to PD-1 leads to the inhibition of cytotoxic T-cells [17]. By targeting tumors enriched with TILs that express PD-L1, T-cells within the tumor microenvironment (TME) can be activated [18]. Interestingly, higher rates of PD-L1 expression were found in TNBC patients than with other types of breast cancers [19]. Increased PD-L1 expression can occur via amplification of the PD-L1 gene at 9p24.1. While PD-L1 is normally amplified by 0.7% across most human cancers, this was elevated to 2.0% in TNBC and HER-2-positive breast cancers [20,21]. Sabatier et al. also demonstrated that higher PD-L1 expression resulted in a 50% pathological complete response (pCR), while normal PD-L1 expression resulted in a 21% pCR in response to neoadjuvant chemotherapy [22].

The anti-PD-L1 antibody Atezolizumab has been extensively studied and tested as first-line therapy in a phase I clinical trial (NCT01375842). Women with metastatic TNBC exhibited a median progression-free survival (PFS) of 4.0 months (95% CI, 1.6–10.1), a median overall survival (OS) of 17.6 months (95% CI, 10.2–N/A), and an incidence of treatment-related adverse events (trAEs) of 62% [23]. Contrarily, women who received Atezolizumab as second- or third-line therapy exhibited a median PFS of 1.8 months (95% CI, 1.4–2.3), a median OS of 7.3 months (95% CI, 6.1–10.8) and an incidence of trAEs of 43% [23]. The subsequent IMpassion130 phase III trial (NCT02425891) investigated whether Atezolizumab, combined with the chemotherapeutic agent nab-Paclitaxel, would generate improved clinical outcomes relative to chemotherapy alone. Blockade of PD-1/PD-L1 interactions in untreated metastatic TNBC patients significantly reduced tumor growth [24]. The median PFS prolongated to 7.2 months from 5.5 months (HR, 0.80; 95% CI, 0.69 to 0.92; *p* = 0.002) in the intention-to-treat population (with patients being randomized according to randomized treatment), and to 7.5 months from 5.0 months (HR, 0.62; 95% CI, 0.49 to 0.78; *p* < 0.001) among patients with PD-L1^+^ tumors [24]. Furthermore, the median OS prolongated to 21.3 months from 17.6 months (HR, 0.84; 95% CI, 0.69 to 1.02; *p* = 0.008) in the intention-to-treat population, and to 25.0 months from 15.5 months (HR, 0.62; 95% CI, 0.45 to 0.86) among patients with PD-L1^+^ tumors [24]. As such, Atezolizumab in combination with nab-Paclitaxel was FDA-approved for the treatment of locally advanced or metastatic PD-L1^+^ TNBC in March 2019.

Similarly, the anti-PD-1 antibody Pembrolizumab was assessed in the KEYNOTE-086 phase II trial (NCT02447003), administered as a monotherapy to cohorts of previously treated metastatic TNBC and untreated PD-L1^+^ metastatic TNBC [25]. The median PFS was 2.0 months (95% CI, 1.9 to 2.0) in the previously treated population and 2.1 months (95% CI, 2.0 to 2.2) for the patients with PD-L1^+^ tumors [25,26]. The median OS was 9.0 months (95% CI, 7.6 to 11.2) in the previously treated population and 18.0 months (95% CI, 12.9 to 23.0) for the patients with PD-L1^+^ tumors [25,26]. The incidence of trAEs was 60.6% in the previously treated population and 63.1% in the PD-L1^+^ population [25,26], comparable to anti-PD-L1 monotherapy. This study concluded that Pembrolizumab showed durable anti-tumor activity for patients with PD-L1^+^ metastatic TNBC and was followed by the KEYNOTE-335 phase III trial (NCT02819518). This study investigated whether Pembrolizumab, in combination with various chemotherapeutic agents, would provide improved clinical outcomes relative to chemotherapy with placebo. The results indicated that Pembrolizumab plus chemotherapy increased the median PFS from 5.6 months (95% CI, 5.3–7.5) to 9.7 months (95% CI, 7.6–11.3), relative to chemotherapy alone [27]. The objective response rate (ORR) increased from 40% (95% CI, 30%–50%, chemotherapy alone) to 53% (95% CI, 46–60) for the combinatorial treatment [27]. Fatal adverse events occurred in only 2.5% of patients receiving the combinatorial treatment, which was discontinued in 11% of patients due to trAEs [27]. As such, Pembrolizumab in combination with chemotherapy was granted accelerated FDA approval for patients with locally recurrent, unresectable, or metastatic TNBC in November 2020.

Conversely, several reports showed that anti-PD-1 or anti-PD-L1 antibodies did not exhibit better outcomes than chemotherapy in TNBC, while contributing to neurotoxicity [28,29]. Atezolizumab monotherapy demonstrated a low PFS of 1.9 months (95% CI, 1.4–2.5) and a high incidence of trAEs at 68% [23], leading to questions regarding its clinical efficacy. In the IMpassion130 trial, only a subset of patients with PD-L1^+^ tumors benefited from the treatment compared to the intention-to-treat population (Table 1). Despite the importance of PD-L1 and TILs as predictive biomarkers, a clinical method for TIL assessment needs to be standardized, which may impact patient outcomes of ICIs [30]. Furthermore, the IMpassion130 trial failed to incorporate an Atezolizumab monotherapy arm, whereas the IMpower110 phase III clinical trial (NCT02409342) compared single-agent Atezolizumab to chemotherapy (Table 1). Preliminary results showed a modest increase in median OS from 14.1 months with chemotherapy to 17.5 months with Atezolizumab [31]. Results from the recent IMpassion131 phase III trial (NCT03125902) showed ineffectiveness in the combination of Atezolizumab and chemotherapy for the patients with metastatic TNBC. The median PFS increased insignificantly from 5.7 months (95% CI, 5.4–6.5) to 6.0 months (95% CI, 5.6–7.4), and the median OS increased slightly from 22.1 months (95% CI, 19.2–30.5) to 22.8 months (95% CI, 17.1–28.3) in the placebo plus Paclitaxel and Atezolizumab plus Paclitaxel treatment arms, respectively [32]. Another concern with the IMpassion130 trial is that patients previously treated with adjuvant chemotherapy and relapsed within 12 months were excluded from the trial. It is expected that this will be clarified in the IMpassion132 phase III trial (NCT03371017), which is studying an anti-PD-1 and chemotherapy combinatorial treatment [33]. With respect to Pembrolizumab, the KEYNOTE-119 phase III clinical trial compared single-agent Pembrolizumab to chemotherapy in metastatic TNBC patients (Table 1). The median PFS decreased from 3.3 months (95% CI, 2.7–4.0) with chemotherapy to 2.1 months (95% CI, 2.0–2.1) with Pembrolizumab monotherapy [34]. The median OS also decreased from 10.8 months (95% CI, 9.1–12.6) with chemotherapy to 9.9 months (95% CI, 8.3–11.4) with Pembrolizumab monotherapy, concluding that Pembrolizumab monotherapy did not provide clinical improvements relative to chemotherapy [34]. With conflicting trial results regarding the clinical efficacy of Pembrolizumab, further research is warranted. In addition, researchers have reported associations between ICI monotherapy and immune-mediated neurotoxicity due to potential molecular mimicry or hidden autoimmunity with neuronal antigens in the peripheral nervous system [29,35,36]. As such, improvements to PD-1/PD-L1 axis-associated immunotherapy in TNBC are needed.

### 2.2. CTLA-4 and Dual Checkpoint Inhibition

The cytotoxic T lymphocyte-associated protein 4 (CTLA-4) is an inhibitory receptor, constitutively expressed on regulatory T cells (T_reg_) and upregulated on the surface of activated CD4^+^ and CD8^+^ T-cells [37,38]. CD-80/B7-1 and CD-86/B7-2 ligands expressed on antigen-presenting cells (APCs) can bind to CD-28 on T-cells to induce T-cell activation and cytokine secretion [39]. CTLA-4 competes with CD-28 to bind CD-80/B7-1 and CD-86/B7-2 ligands, and negatively regulates T-cell function [37,38,39,40]. CTLA-4 is essential for neutralizing potential naïve autoreactive T-cells in the secondary lymphoid organs [40]. The inhibition of CTLA-4 can prevent T-cell inhibition and enhance T-cell anti-tumor activity, making it an attractive target for antibody-based therapy (Figure 1). The anti-CTLA-4 antibody Ipilimumab, in combination with the anti-PD-1 antibody Nivolumab, has been FDA-approved for the treatment of melanoma, lung cancer, and renal cell carcinoma, among other cancers [41,42]. The CheckMate032 phase 1/2 trial (NCT01928394) showed modest improvements in ORR for non-small cell lung cancer, from 10% of patients receiving Nivolumab alone to 33% of patients receiving Nivolumab plus Ipilimumab in combination [43]. More recently, the CheckMate-9LA phase III trial (NCT03215706) demonstrated the superior clinical effectiveness of Nivolumab and Ipilimumab in combination with chemotherapy for metastatic or recurrent non-small-cell lung cancer. Combinatorial treatment increased the median PFS from 5.0 months to 6.7 months, increased the median OS from 10.9 months to 15.6 months, and slightly increased the incidence of trAEs from 38% to 47% relative to chemotherapy alone [44].

Since anti-PD-1/PD-L1 monotherapy was shown to benefit only a subset of the population, additional clinical trials combining anti-CTLA-4 monotherapy have been performed in an effort to improve clinical outcomes but failed to do so. A randomized phase II trial (NCT02519322) testing the clinical efficacy of the Nivolumab as monotherapy in combination with the anti-CTLA-4 antibody Ipilimumab in patients with high-risk resectable melanoma was terminated early because of the high incidence of trAEs. Grade 3 trAEs were reported in 8% of patients in the Nivolumab monotherapy treatment arm and 73% of patients in the combinatorial treatment arm [45]. In a separate phase II clinical trial (NCT02536794), the anti-PD-1 antibody Durvalumab and anti-CTLA-4 antibody Tremelimumab were combined in a single treatment arm without comparison to monotherapy. The drugs were administered to estrogen receptor-positive (ER^+^) breast cancer and TNBC patients. Preliminary data suggested that clinical benefit was derived in 71% of TNBC patients, but in none of the ER^+^ breast cancer patients [46]. This trial was also discontinued due to ORRs not meeting the required criteria. The termination of both trials suggested that the risks associated with dual immune checkpoint inhibition may not exceed the benefits.

The use of anti-CTLA-4 antibodies in monotherapy and in combination with anti-PD-1/PD-L1 antibodies have been discredited, due to the lack of significant clinical response and high incidence of autoimmunity. Its ORR in combination with Nivolumab was modest relative to the results seen in the trial by inhibition of the PD-1/PD-L1 axis. Alternatively, anti-CTLA-4 antibodies may be complemented by the stimulator of interferon genes (STING) agonists that promote intratumoral T-cell infiltration and sensitize tumor cells to NK cell killing [47,48]. Harding et al. reported minimal tumor regression following anti-CTLA-4 therapy in B16 murine melanoma models with STING knockout [49]. However, CTLA-4 antibodies may lead to autoimmunity, due to its role in maintaining self-tolerance [50,51]. In a study by Tivol et al., CTLA-4 ^−/−^ mice demonstrated excessive proliferation of the lymph nodes, severely destructive myocarditis and pancreatitis, suggesting the role of CTLA-4 in deleting autoreactive T-cells in the periphery [52]. Consistent with this, a study by Gough et al. showed that polymorphisms within the human CTLA-4 gene are associated with autoimmune diseases [53]. This was also consistent with the CheckMate238 clinical trial (NCT02388906), in which grade 3 or 4 trAEs were reported among 45.9% of participants in the Ipilimumab group, followed by discontinued treatment for 42.6% of the patients [54,55]. Interestingly, the efficacy of immune checkpoint inhibition was found to depend on the composition of commensal bacteria. Introducing strains such as *Bacteroides fragilis* to germ-free or antibiotic-treated mice helped to overcome the poor response of CTLA-4 blockades and further induced polarization of T helper cell 1 (T_H1_) [56]. As such, anti-CTLA-4 alone has not been considered as a viable front-line treatment option for TNBC to date.

### 2.3. Next Generation Immune Modulatory Targets

Many immune checkpoint molecules beyond PD-1/PD-L1 and CTLA-4 are currently under clinical investigation to identify additional drug targets for PD-L1^-^ and TIL^−^ patients or to enhance ICI monotherapy. The immunosuppressive protein sialic acid binding Ig-like lectin-15 (Siglec-15), normally expressed on myeloid cells, was shown to be upregulated in many human cancers [57]. With similar structural homology to PD-L1, Siglec-15 is targeted using the anti-Siglec-15 monoclonal antibody, NC318 [58]. In the phase I/II clinical trial (NCT03665285), NC318 was administered to patients with advanced or metastatic solid tumors, with results expected in 2021. T-cell Ig and ITIM domain (TIGIT) is another inhibitory receptor expressed on lymphocytes and upregulated upon activation [59]. Its ligands include CD112, CD113, and CD155, all of which are over-expressed in TNBC [60,61,62]. Interestingly, pre-clinical experiments on TIGIT^−/−^ mice suggested higher safety and fewer trAEs than anti-PD1/PD-L1 or anti-CTLA-4 monotherapies [63]. Lymphocyte-activation gene 3 (LAG-3) and T-cell Ig and mucin domain-containing protein 3 (TIM-3) are also attractive immunosuppressive targets being actively investigated in breast cancer. Saleh et al. reported that the co-inhibition of PD-1 and PD-L1 further upregulated LAG-3 and TIM-3 in T-cells and T_regs_ when co-cultured with TNBC cells, but not with other breast cancer cell lines [64].

In contrast to co-inhibitory immune molecules, co-stimulatory molecules are equally attractive targets in immunotherapy. OX40 is a positive immune checkpoint molecule involved in T-cell proliferation following activation, and T_reg_ suppression [65]. Several clinical trials are investigating the efficacy of anti-OX40 antibodies in combination with ICI monotherapy for TNBC patients (NCT02528357, NCT03971409, NCT03241173). The inducible co-stimulatory receptor 4-1BB is expressed on activated T-cells and NK cells, and can be exploited to improve anti-tumor immunity [66,67]. However, the therapeutic value of 4-1BB in TNBC patients remains open to investigation. The glucocorticoid-induced TNFR-related (GITR) and inducible co-stimulator of T-cells (ICOS) are also attractive stimulatory targets. The ICOS monoclonal antibody, JTX-2011, was administered to TNBC patients in the phase I/II clinical trial (NCT02904226). Preliminary results reported a disease control rate of 25% with JTX-2011 monotherapy, and 29% in combination with Nivolumab (anti-PD-1) [68]. Notably, two grade 5 trAEs were observed among patients in the combinatorial treatment arm, potentially due to the simultaneous expression of ICOS on immunosuppressive T_regs_ [68]. In this regard, agonist antibodies in immunotherapy have been approached with caution and require additional research before implementation in the clinic.

## 3. Factors Affecting the Efficacy of Immune Checkpoint Inhibitors

Blockade of PD-1/PD-L1 and/or CTLA-4 has been demonstrated to be effective and durable in certain types of cancers. However, fewer than 10% of patients respond to single-agent treatments [69]. Co-administration of ICIs with chemotherapeutic agents, as described above, contributes to the enhanced immune priming [24,27,70]. Combinations with other therapies or factors may also increase efficacy, as described below.

### 3.1. Dysregulated Tumor Vasculature

Poor clinical outcomes associated with TNBC are partly attributed to the dysregulated angiogenesis that results in hypoxic conditions within the TME. As tumors expand over time, tumor cells within the tumor core become increasingly hypoxic, such that there is an upregulation of angiogenic growth factors associated with the expression of hypoxia-induced transcription factor (HIF-1) [71]. Angiogenic growth factors including vascular endothelial growth factor (VEGF), endothelial growth factor (EGF), and platelet-derived growth factor (PDGF) promote the migration of endothelial cells towards the tumor core, through which tumors acquire nutrients for growth and a route to metastasize into systemic circulation [71,72,73]. Under normal conditions, angiogenic growth factors are balanced by the metabolic demands of the surrounding tissue. However, the hypoxic conditions of the TME hijack this balance in favor of dysregulated angiogenesis [72,73].

Tumor-associated capillaries contribute to immunosuppression by reducing trafficking and activation of effector T-cells and restricting entry of cytotoxic drugs [74,75]. Tumor-associated endothelial cells may release interferon-γ (IFNγ) that upregulates PD-L1 expression to inhibit the anti-tumor activity of T-cells [76]. This is consistent with a study by Kammertoens et al., who showed that intratumoral injection of IFNγ caused rapid loss of tumor-associated vessels but also impeded anti-tumor activity of effector T-cells [77]. As IFNγ is mainly expressed by activated infiltrating T-cells, the IFNγ-mediated upregulation of PD-L1 can be exploited in a combinatorial therapy for non-responders to anti-PD-1 monotherapy [78]. Tian et al. reported an increase in pericyte coverage and a decrease in pulmonary metastasis (indicators of vasculature normalization) following immune checkpoint blockade in mice bearing TNBC 4T1 tumors [79,80].

To inhibit the growth and metastasis of TNBC and promote the anti-tumor activity of effector T-cells, a combination of immunotherapy with anti-angiogenic factors has been investigated. In the IMbrave150 phase III clinical trial (NCT03434379), the anti-PD-1 agent Atezolizumab was combined with anti-angiogenic agent Bevacizumab and compared with protein kinase inhibitor Sorafenib alone (Table 2). There was a clinically significant improvement in median PFS, which was 6.8 months (HR, 0.58; 95% CI, 5.7–8.3) in the Atezolizumab/Bevacizumab group and 4.3 months (HR, 0.59; 95% CI, 4.0–5.6) in the Sorafenib group [81]. Of note, grade 3 or 4 trAEs occurred in 56.5% of patients receiving Atezolizumab/Bevacizumab and 55.1% of patients receiving Sorafenib [81]. Furthermore, Huang et al. showed that lower doses of anti-VEGFR2 antibody improve tumor-associated vessel perfusion and reduce tumor hypoxia more effectively than the immunoglobulin-G (IgG) control and high-dose anti-VEGFR2 treatment groups [82]. This highlights the importance of further examining dosage and timing to optimize the combinatorial efficacy of anti-angiogenic and ICI treatments. The excessive use of anti-angiogenic agents may impede drug delivery and limit the infiltration of effector T-cells in the tumor [82,83]. Wu et al. proposed using angiopoietin-2 as a biomarker in addition to as a therapeutic target for predicting the clinical outcome of Bevacizumab monotherapy, due to its important role in treatment resistance [84]. Therefore, the combination of ICI with anti-angiogenesis therapy may represent a promising avenue for the future of TNBC treatment.

### 3.2. Interleukin-8 and CXCR1/CXCR2

**Figure 2:**
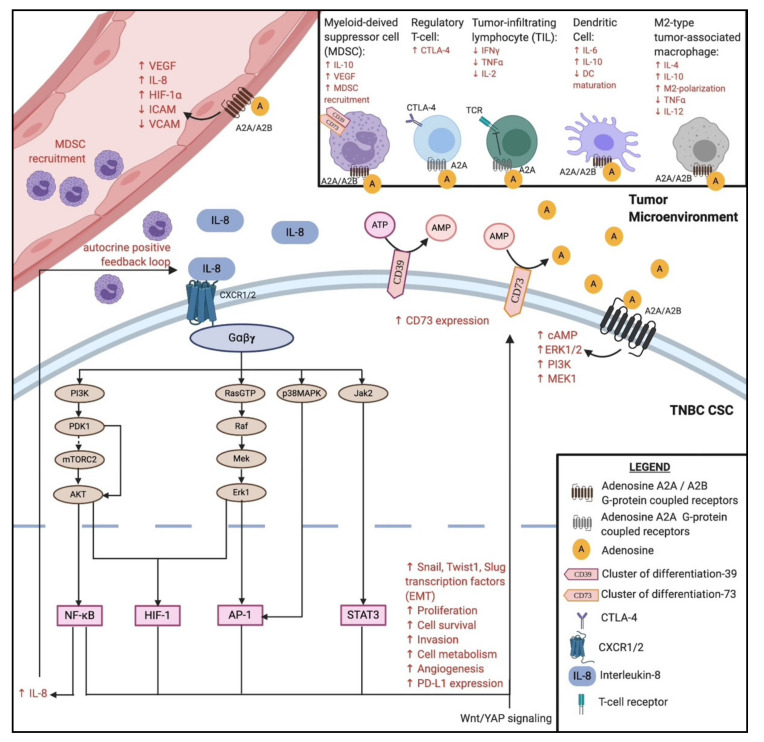
Various tumor-promoting mechanisms that can be exploited in combination with immune checkpoint inhibition. Overexpression of IL-8 and CXCR1/2 in TNBC generates an autocrine positive feedback loop that promotes EMT, HIF-1-mediated angiogenesis and endothelial migration, and recruitment of immunosuppressive MDSCs to the tumor microenvironment (TME). Transcription of NF-κB, HIF-1, AP-1, and STAT3, in combination with Wnt and YAP signaling, upregulates CD73 on TNBC cells. Immunogenic cell death in the TME releases ATP in abundance, which is subsequently converted to adenosine by CD39 and CD73. Excess adenosine in the TME bind to adenosine type 1 purinergic G-protein coupled receptors that further facilitate MDSC expansion, CTLA-4 upregulation, inhibition of TIL activation, inhibition of DC maturation, and M2-type TAM polarization. Activation of adenosine receptors on tumor-associated endothelial cells promotes angiogenesis via upregulation of VEGF and HIF-1α, along with a simultaneous downregulation of adhesion molecules essential for diapedesis. Finally, activation of adenosine receptors on the TNBC cell further upregulate cytosolic cyclic AMP (cAMP), Erk1/2, PI3K, and MEK1, all of which further enhance the IL-8 signaling pathway. Figure created with BioRender.com.

Interleukin-8 (IL-8) is a chemokine responsible for the recruitment of neutrophils to areas of inflammation, infection, or injury [90]. IL-8 is secreted by macrophages, epithelial cells, airway smooth muscle cells, and endothelial cells [90,91]. IL-8 binds to CXCR1 and CXCR2 G-protein coupled receptors on granulocytes, monocytes, and endothelial cells [90,91,92]. Interestingly, breast cancer patients that highly expressed IL-8 were associated with poor relapse-free, overall, and distant metastasis-free survival [92]. Cheng et al. reported an overexpression of IL-8, CXCR1, and CXCR2 in breast, prostate, lung, and colon cancers [93]. The binding of IL-8 to CXCR1/CXCR2 was shown to induce the transition from an epithelial-like to mesenchymal-like status, thus promoting the migration, invasion, and reconstitution of a secondary tumor [94,95]. CXCR2 signaling also promotes the migration of human endothelial cells and angiogenesis, forming a positive feedback loop to further promote epithelial-to-mesenchymal transition (EMT) [96,97]. IL-8 inhibitors might play an important role as anti-angiogenic agents in combination with ICIs. In addition, IL-8 signaling directly promotes immunosuppression in the TME via the recruitment of myeloid-derived suppressor cells (MDSCs, Figure 2). MDSCs are capable of depleting nutrients such as L-arginine, L-tryptophan, and L-cysteine, all of which are essential for T-cell expansion [98,99]. MDSCs also inhibit anti-tumor activity by producing reactive oxygen species and peroxynitrite that can directly inactivate T-cell receptors [100], and by producing reactive nitrogen species that hinder the infiltration of cytotoxic T-cells into the tumor core [101]. Highfill et al. showed that early treatments with anti-PD-1 agents prevented tumor growth, but late treatments showed less benefit due to the presence of MDSCs in the TME [102]. They further showed that anti-CXCR2 monoclonal antibody therapy led to significant anti-tumor activity, even after delayed anti-PD1 treatment [102]. Sanmamed et al. also suggested using IL-8 as a prognostic biomarker to predict the clinical benefit of ICI therapy [103]. They observed that serum IL-8 levels decreased significantly among patients responding to anti-PD-1 checkpoint inhibition (*p* < 0.001) [103]. Serum IL-8 levels also increased significantly among non-responders to anti-PD-1 blockade (*p* = 0.013) [103]. Together, these results suggest that IL-8 inhibition may represent a potential candidate for combinatorial therapy with ICIs.

The anti-IL-8 antibody HuMax-IL8 (also known as BMS-986253) was developed for the successful depletion of tumor-secreted IL-8 and inhibition of CSC mesenchymal properties [85]. HuMax-IL8 also reduced the recruitment of polymorphonuclear MDSCs to the tumor core by preventing IL-8 from binding to CXCR2 receptors on the MDSCs [85]. The phase I trial for HuMax-IL8 (NCT02536469) concluded that serum IL-8 was significantly reduced after the third day of treatment relative to control (*p* = 0.0004) [86]. The incidence of trAEs was 33%, which was much lower than that of anti-angiogenic agents observed in clinical trials [86]. Additionally, the MAGIC-8 phase Ib/II clinical trial (NCT03689699) combined the anti-PD-1 agent Nivolumab with HuMax-IL8 for the treatment of hormone-sensitive prostate cancer [104]. The results of this study are expected in 2022. Thus, IL-8 inhibition may provide a benefit among non-responders of ICIs in TNBC patients, warranting further exploration.

### 3.3. CD73 Expression

Cluster of differentiation 73 (CD73), normally expressed on T_reg_ cells, is an ectonucleotidase that dephosphorylates extracellular AMP to adenosine [105]. Its expression on bulk tumor cells and mesenchymal-like CSCs generates excess adenosine in the TME, which binds to the adenosine type 1 purinergic G-protein coupled receptor family (denoted A1, A2A, A2B, and A3), some of which are involved in inhibiting effector T-cell responses [106,107]. Adenosine acting on the A2A receptors has a suppressive effect on effector T-cells and an obligatory role in tumor immunomodulation [105,106,108]. In conjunction with IL-8 signaling, the excess adenosine levels facilitate MDSC expansion in the TME via the activation of A2B receptors to enhance immunosuppression [109]. Jin et al. showed that the knockdown of tumor CD73 and subsequent transfer of tumor-specific T-cells significantly enhanced tumor-free survival in tumor-bearing mice [108]. Hypoxic conditions within the tumor core, along with Wingless (Wnt) signaling, upregulate CD73 expression [110,111]. In addition to increasing immunosuppressive adenosine levels in the TME, CD73 modulates cell adhesion molecules within the endothelium, whereas upregulated CD73 promotes the attachment of lymphocytes and reduces their migration into the lymph nodes [112]. CD73 overexpression is also associated with poor prognosis in TNBC [113], highlighting the potential for targeting CD73 as part of a combinatorial treatment (Figure 2). In the phase I trial (NCT02754141), it was shown that the anti-CD73 agent BMS-986189 in combination with the anti-PD-1 antibody Nivolumab was well-tolerated in patients with advanced solid tumors [87]. TrAEs were observed in 58% of the patients receiving the combination, of which 15% were grade 3 in nature [87]. Alternatively, the SYNERGY phase I/II clinical trial (NCT03616886) compared the anti-CD73 agent Oleclumab with the anti-PD-1 agent Durvalumab, and chemotherapeutic agents, in single and combinatorial treatment arms (Table 2). The results of this study are expected in 2022.

### 3.4. Long Non-Coding RNAs and Microsatellite Instability

Long non-coding RNAs (lncRNAs) are ~200 nucleotides long and do not code for protein products [114]. Their role in disease regulation was only brought to light recently, when the lncRNA urothelial carcinoma-associated 1 (UCA1) was shown to contribute to resistance against tamoxifen therapy in ER^+^ breast cancers [115]. The lncRNA ROR was associated with a decrease in the expression of E-cadherin, an epithelial marker, and an increase in the expression of the mesenchymal markers vimentin, zeb1, and zeb2 [116]. ROR promoted metastasis via the EMT process and contributed to tamoxifen therapy resistance [116]. LncRNAs (e.g., the nuclear-enriched autosomal transcript1 (NEAT1)) were also associated with immunosuppression. Yan et al. reported that NEAT1 inhibition suppressed CD8^+^ T-cell apoptosis and enhanced anti-tumor activity [117]. Metastasis-associated lung adenocarcinoma transcript1 (MALAT1) is another lncRNA that was observed to upregulate the expression of PD-1 and CD-47 [118]. Wang et al. showed that knock-down of MALAT1 by shRNA decreased the expression of PD-1, and also suppressed the EMT process [119]. Despite the growing evidence regarding the potential role of lncRNAs in immunotherapy resistance, targeting them in the clinic remains a challenge. LncRNAs would be considered in combinatorial therapy, however, targeting them through RNAi-mediated gene silencing therapy, antisense oligonucleotide-based therapy, or small molecule inhibitors remains expensive and inconvenient, defies precision medicine, and may contribute to unforeseen systemic adverse events or trAEs [120,121]. Further research is therefore required before investigating their potential in the clinic.

Microsatellite instability (MSI) status has also been considered to impact efficacy of ICIs and is therefore used as a reliable biomarker. Microsatellites are short repetitive sequences scattered throughout the human genome as a result of aberrant DNA mismatch repair mechanisms [122]. Recent reports have suggested that a high MSI status, indicative of a hypermutation phenotype, may sensitize patients to ICIs [123,124]. Interestingly, 6.9% of TNBC cases showed a complete loss of relevant mismatch repair proteins, which were correlated with significantly greater PD-L1 expression [125]. Other contributors to MSI status may include tumor mutational burden, which is a measure of nonsynonymous mutations in tumor cells [126], and BRCA1 mutation status. BRCA1 expression modulates the silencing mechanisms in satellite DNA, such that BRCA1-mutated TNBC exhibit higher microsatellite instability than BRCA1-wildtype TNBC [127,128]. The phase II trial (NCT01876511) comparing Pembrolizumab efficacy in MSI-positive and MSI-negative colorectal cancer patients demonstrated an immune-related PFS of 78% among MSI-positive patients and 11% among MSI-negative patients [129]. Pembrolizumab was subsequently FDA-approved for unresectable or metastatic solid tumors with high MSI.

### 3.5. Wnt and YAP Signaling

Many components of the Wnt signaling pathway are involved in tumorigenesis via EMT and tumor regeneration [130,131,132,133]. Cytoplasmic stabilization and nuclear translocation of β-catenin into the nucleus allows for the expression of Wnt target genes [132]. The enrichment of β-catenin, nuclear translocation, and dysregulated Wnt signaling are all associated with poor clinical outcomes of TNBC [134]. The inhibition of Wnt signaling leads to the suppression of CSCs and bulk tumor cells in both TNBC cell lines and patient-derived xenograft (PDX) models [135]. Additionally, activation of β-catenin in tumor cells prevents spontaneous T-cell priming and infiltration of effector T-cells into the TME [136]. A mouse model for hepatocellular carcinoma also demonstrated the role of β-catenin in immune escape and resistance to anti-PD-1 monotherapy [137]. This was consistent with a study by Castagnoli et al., which reported a strong correlation between downstream Wnt signaling effector expression and PD-L1 expression in TNBC [138]. Furthermore, several studies have shown that Wnt inhibitors reduce PD-L1 expression and Wnt agonists enhance PD-L1 expression, suggesting Wnt inhibitors as possible adjuvants to anti-PD-1, anti-PD-L1, or anti-CTLA4 therapies [138,139]. In addition to modulating PD-L1 expression, Wnt/β-catenin signaling has been shown to inhibit T-cell maturation and activation and inhibit dendritic cells (DCs) from secreting chemokines essential for T-cell activation [140,141,142].

Yes-associated protein (YAP) signaling has also been shown to contribute to the EMT process. A study by Cordenonsi et al. demonstrated a strong correlation between YAP expression and mesenchymal-like CSC surface markers [143,144]. Moreover, YAP knockdown in TNBC cell lines led to a loss of cell proliferation and invasiveness [145]. Besides the role of YAP in modulating Wnt signaling via the β-catenin destruction complex [146], YAP was also shown to directly modulate PD-L1 expression in human TNBC [147]. Interestingly, recent studies have reported that the major transcription factor in YAP signaling, TEAD, has a binding site located close to the PD-L1 promoter. As such, YAP can bind directly to the enhancer region of PD-L1 [148,149]. YAP signaling has also been implicated in recruiting MDSCs to the TME, contributing to the suppression of anti-tumor activity in a similar manner to IL-8 [150]. Moreover, YAP signaling regulates the recruitment of tumor-associated macrophages (TAMs) to the TME. TAMs can differentiate into M1 and M2 phenotypes. M1-type TAMs secrete pro-inflammatory interleukin-1 (IL-1) and tumor necrosis factor-α (TNFα), which promote the expression of inducible nitric oxide synthase (iNOS) to enhance the antigen presentation process [151]. In contrast, M2-type TAMs secrete IL-10, IL-4, arginase-1, and other cytokines involved in resolving inflammation, wound-healing, and facilitating tumor growth [151]. High transcriptional levels of YAP have been reported to disproportionately promote the expression of M2-type TAMs in the TME to enhance tumor growth, drug resistance, and metastasis [152,153].

Wnt and YAP signaling might be important pharmacological targets for eliminating mesenchymal and epithelial CSC populations while simultaneously enhancing ICI immunotherapy outcomes. However, it remains unclear whether they can be implemented in combinatorial treatments, as molecular mechanisms remain unclear. Furthermore, the complexity of Wnt signaling and its broad involvement in normal stem cell self-renewal, differentiation, and organ homeostasis [154] suggest that inhibiting this pathway may be counterproductive and yield intolerable toxicity. However, a phase I clinical trial (NCT01302405) using the Wnt signaling inhibitor PRI-724 concluded an acceptable toxicity profile, with 11% of patients experiencing grade 3 trAEs [88]. Inhibition of YAP signaling poses its own challenges, as Ni et al. reported that YAP is essential for the differentiation of T_reg_ cells, which prevent autoimmune disease [155]. Our recent report suggested that dual inhibition of Wnt and YAP signaling, but neither alone, is required for suppressing both mesenchymal and epithelial CSC populations and diminishing Paclitaxel-induced CSC enrichment in immune-deficient mice [156]. Although combination therapies typically result in better drug responsiveness and synergism than single-agent monotherapies [157], further studies are needed to clarify the efficacy and toxicity of combination therapy with Wnt and/or YAP inhibitors and ICIs.

### 3.6. Nanoparticle Platforms as a Delivery System

Traditional ICIs are delivered systemically as monoclonal antibodies, which have the potential of activating self-reactive T-cells [158,159]. Immune-related adverse effects may be amplified in combinatorial ICI therapies and may be reduced or resolved by the use of corticosteroids. However, this can increase the risk of other complications and diminish the therapeutic potential of ICIs [158]. Nanoparticle platform-based therapies have revolutionized drug delivery owing to their ability to accumulate in solid tumors, reduce toxicity to vital organs, and increase the therapeutic index [160,161,162]. Studies have demonstrated the ability of nanoparticles (NPs) to deliver both hydrophobic and hydrophilic drugs, small molecule drugs, and antibodies to the TME, with minimal toxicity to surrounding tissues [160,163]. Studies have also demonstrated the ability of NPs to efficiently interact with and activate dendritic cells and macrophages within the TME [164]. For example, synthetic high-density lipoprotein (sHDL) nanodiscs loaded with Doxorubicin and combined with anti-PD-1 ICIs resulted in a seven-fold increase in IFNγ-positive CD8^+^ T-cells in the TME, compared with Doxorubicin treatment alone [165]. Additional benefits of the sHDL nanodiscs included the complete regression of colon carcinoma tumors in 80–88% of the treated animals and no apparent cardiotoxicity post-treatment [165]. In another report, poly(lactic-co-glycolic acid) (PLGA) NPs co-loaded with Paclitaxel, combined with detoxified bacterial lipopolysaccharide, resulted in a significant increase in T_H1_ cells in B16F10 mouse models, in comparison to Paclitaxel treatment alone [166]. PLGA NP-treated mice exhibited a 40% lower tumor volume and a higher degree of retention in biological activities of both co-encapsulated drugs [166]. In a third study, nanoscale coordination of polymer (NCP) core-shell nanoparticles co-encapsulated with Oxaliplatin and pyrolipids for photodynamic therapy demonstrated synergism with anti-PD-1 ICI therapy in CT26 and HT29 mouse models [167]. This treatment also reduced tumor volume to 2.9% of their original size when combined with anti-PD-1 therapy, and to 39.1% of their original size in the absence of anti-PD-1 therapy [167]. Such synergy with anti-PD-1 therapy was also observed with peptide-based structure-transformable NPs [168], dendrimers used for siRNA delivery [169], and inorganic NPs composed of gold, titanium dioxide, or iron oxide [170,171]. It is evident that various NP platforms can be used to enhance tumor immunogenicity, favor ICI therapies, and promote the pharmacokinetics and pharmacodynamics of combinatorial treatments.

## 4. Cancer Cell Antigens: Potential Therapeutic Targets

Since ICI immunotherapy for TNBC benefits only a subset of patients, developing treatment strategies for TNBC patients with lower immunogenic tumors remains an unmet medical need. An ideal therapy would be one with target antigens expressed on bulk tumor cells and also overexpressed in CSC populations [172]. To effectively boost anti-tumor immunity, antigen expression should be evaluated on both CSCs and bulk tumor cells. Interestingly, cancer-testis antigens (CTAs) have been shown to be overexpressed in CSC populations [173]. CTAs are normally expressed in germ-line tissues such as the testis, placenta, and ovaries, but are also highly expressed across several cancer types [174]. In the following section, we will describe some CTAs and two other tumor antigens that have shown potential as biomarkers or immunotherapeutic targets in TNBC.

### 4.1. Cancer-Testis Antigens

The progression from primary tumor to metastasis is somewhat resembled in the gonads, where trophoblasts invade and burrow into the endometrium [175]. Placenta-specific protein 1 (Plac1) normally plays an important role in trophoblast invasion and migration but is also found to be expressed in a large range of human cancers [176,177]. As such, trophoblast-specific pathways could be reactivated, contributing to the activation of lymphocyte-mediated tumor growth [177]. It has been hypothesized that placental mammals have a certain degree of placental invasiveness that is positively correlated with the incidence of metastatic tumors [178]. Females with a lower degree of placental invasiveness in some species have evolved mechanisms to counter trophoblast invasion, and thus cancer metastasis [179]. Koslowski et al. showed that siRNA inhibition of Plac1 effectively suppressed tumor migration and invasion in breast cancer cell lines [180]. However, the correlation between Plac1 expression and clinical prognosis of TNBC remains unknown [180], and more research is required.

Another strong CTA candidate for TNBC immunotherapy is the New York esophageal squamous cell carcinoma 1 (NY-ESO-1). NY-ESO-1 is normally expressed in primary spermatocytes and rapidly declines in female oogonia [181]. NY-ESO-1 expression is believed to be involved in the proliferation of stem cells and epithelial CSC populations [182]. Ademuyiwa et al. reported NY-ESO-1 expression in 16% of TNBC patients, and antibody responses against NY-ESO-1 were observed in 73% of TNBC patients who were NY-ESO-1-positive [183]. It was also reported that NY-ESO-1-positive patients had higher CD8^+^ T-cell infiltration in TNBC tumors [184]. NY-ESO-1-specific CD8^+^ T-cells showed upregulated PD-1 expression, suppressing anti-tumor immunity [184]. As such, NY-ESO-1 may be an attractive candidate for a combinatorial therapy with anti-PD-1 ICI.

The MAGE-A family is also one of the CTAs that renders TNBC highly immunogenic. Raghavendra et al. reported MAGE-A expression in 47% of TNBC cases, and the majority of NY-ESO-1-positive TNBC tumors were also MAGE-A-positive [185]. MAGE-A is normally involved in chromosomal alignment and centrosome duplication [186]. In TNBC, the expression of MAGE-A, however, was positively correlated with the expression of mesenchymal-like CSC markers such as vimentin, but negatively correlated with the expression of epithelial-like CSC markers such as E-cadherin and β-catenin [187]. Targeting MAGE-A would likely enhance anti-tumor immunity by suppressing EMT. MAGE-A12 has been shown to enhance tumor cell proliferation and CSC maintenance [188]. Since targeting multiple CTAs would provide more benefit than targeting a single CTA in anti-tumor immunotherapy, clinical trials have looked at MAGE-A and NY-ESO-1-based vaccines. However, the MAGRIT phase III trial (NCT00480025, targeting MAGE-A3 in non-small cell lung cancer patients) was terminated due to the lack of clinical benefit [189]. Inter-tumoral heterogeneity could partially explain the extent to which certain CTAs are expressed in TNBC versus other tumors [190]. Further characterization of CTA expression in different types of tumor is essential for developing CTA-based therapies for a given tumor type. Although CTAs represent potential therapeutic targets, their expression remain elusive and appear limited to a small subset of patients [191,192].

### 4.2. Tumor Antigens, Cancer Vaccine, and Oncolytic Virus

The expression of tumor antigens susceptible to immunotherapies in CSC populations have been poorly characterized [186]. While conventional approaches to dendritic cell (DC) vaccines involve using bulk tumor cells as the antigen source, Ning et al. reported that DC vaccines loaded with the lysates of CSCs induced significantly better anti-tumor humoral and cellular immunity than those loaded with bulk tumor cells in mice [193]. Whole-tumor lysates, tumor-antigen-derived peptides, or antigen-encoding RNA/DNA have been used in cancer vaccines [194]. The resultant epitopes from vaccination were presented on major histocompatibility complexes (MHC) I or II by DCs for presentation to CD8^+^ or CD4^+^ T-cells, respectively [195].

Tumor-associated antigens (TAAs) have been considered as a possible solution for targeting CSCs, which are molecules expressed at high levels on cancer cells and low levels on healthy cells [196]. However, TAAs such as gp100 and tyrosinase have the potential for off-target toxicity, due to their systemic expression in normal tissues [196,197]. The challenge to select the appropriate antigen renders tumor vaccinations as a less favorable treatment option. One of the strategies used to overcome this challenge in the clinical setting is to use toll-like receptor (TLR) agonists, to potentiate the innate immune system [198]. In a phase II clinical trial (NCT00960752), the TLR-7/8 agonist Resiquimod was combined with gp100 and MAGE-3 peptide vaccines, with results expected later this year [199]. Other reports showed that TLR-7/8 agonists, among others, increase PD-L1 expression on DCs [200]. More studies will be needed to further consolidate the role of TAAs and CTAs in cancer vaccine and ICI immunotherapy for TNBC.

As an alternative approach to DC vaccinations, oncolytic viruses (OVs) have a well-characterized role in inducing anti-tumor immunity. OVs are naturally or genetically modified vectors that are able to selectively replicate in tumor cells, as tumor cells often have impaired antiviral defenses that make them susceptible to OV infections [201,202,203,204,205,206,207,208]. As OVs replicate in the tumor cells, they trigger an inflammatory response leading to immunogenic cell death (ICD) [204]. Following ICD, damage-associated molecular patterns (DAMPs) are released into the TME, which can be recognized by antigen-presenting cells that secrete cytokines including IFNα, IFNγ, TNFα, IL-6, and IL-12 to recruit innate immune cells [204,205]. Furthermore, ICD results in the release of TAAs and tumor-specific antigens (TSAs) into the TME, which activate antigen-specific CD4^+^ and CD8^+^ T-cells as part of adaptive immunity [206,207]. By stimulating both innate and adaptive immunity, OVs are able to maintain anti-tumor immunological memory to protect against tumor reconstitution. Similar to TLR agonists, a 2017 study revealed that the OV talimogene laherparepvec (T-VEC) increased PD-1 expression [208]. When T-VEC OV therapy was combined with the anti-PD-1 agent Pembrolizumab, the ORR increased by 62% [209]. In a phase I/II trial (NCT02779855), T-VEC OV therapy combined with neoadjuvant chemotherapy was compared with chemotherapy alone. Preliminary data showed an increase in pCR from 30% with chemotherapy alone to 55% in the combinatorial treatment for non-metastatic TNBC patients [210]. As OVs allow for the exploitation of DAMPs and tumor antigens, inflammation induced by the adenovirus primes the tumor for subsequent DC vaccination, which elicits an anti-tumor CD8^+^ T-cell response in mice with lung cancer [211]. Furthermore, the Maraba MG1 rhabdovirus, boosted with adenovirus, led to MAGE-3-specific CD4^+^ and CD8^+^ T-cell expansion that persisted for several months in mice with MAGE-3-positive solid malignancies [212]. Despite some benefits of OVs in immunotherapy, the main challenge is the systemic antiviral mechanism, which has the potential to block OV replication and infection of tumor cells [212].

## 5. Chimeric Antigen Receptor T-Cell Therapy

Chimeric antigen receptor T-cell (CAR-T) therapy involves cytotoxic T-cells that are engineered to express fusion proteins that are capable of recognizing and binding to TAAs expressed by tumor cells. These fusion proteins commonly consist of an extracellular single chain variable fragment (scFv) domain for TAA recognition, a transmembrane domain, and an intracellular T-cell coactivation domain [213]. Engineered CAR-T cells offer personalized immunotherapy but are not subject to the same regulatory signaling as endogenous T-cells [214]. This may contribute to trAEs such as cytokine release syndrome, in which the rapid activation and proliferation of CAR-T cells contributes to the excess production of pro-inflammatory cytokines [215]. Zhou et al. showed that the TAB004 monoclonal antibody, capable of recognizing the tumor variant of mucin1 glycoprotein (tMUC1), can be used to make the scFv domain of their MUC28z CAR-T cells [216]. As tMUC1 is expressed in 95% of malignant tissues (including TNBC); IFNγ levels increased from 2.6 to 18.7 pg/mL among HCC70 cells upon the introduction of MUC28z CAR-T cells [216]. Tumor endothelial marker 8 (TEM8)-specific CAR-T cells have also shown to eliminate TEM8^+^-TNBC tumor cells, and also target tumor-associated endothelial cells [217]. Selection of the right CAR scFv domain dictates the therapeutic potential of CAR-T cells against tumors.

Despite FDA approval, Singh et al. showed relatively poor results of CAR-T therapy against solid tumors, including TNBC, as they are unable to survive in the harsh TME [213]. This has not stopped research groups from exploring CAR-T therapy in TNBC. Based on reports of c-Met overexpression in 52% of TNBC tumors [218], a phase I trial (NCT01837602) demonstrated that c-Met-CAR T-cells did not induce cytokine release syndrome and exhibited on-target effects for c-Met-positive TNBC patients [219]. Previous reports also showed that various TNBC cells lines exhibit moderate to high levels of NKG2D ligand (NKG2DL) [220,221]. Accordingly, the use of the natural killer (NK) cell-activating receptor NKG2D CAR constructs in vivo led to significant MDA-MB-231 tumor regression in mice [220]. Furthermore, a phase I trial (NCT04107142) administered NKG2DL-targeting γ/δ CAR T-cells to patients of varying tumor types, including TNBC. The results for this study are expected in 2021. Similar to the strategies above, further CAR-T research may lead to novel therapeutic options for TNBC.

## 6. Immunotherapy and Metabolism

### 6.1. Metabolic Reprogramming in TNBC

It has long been established that tumor cells exhibit an altered cellular metabolism where they shift their metabolic reliance to sustain their proliferative and competitive needs. Metabolic reprogramming is now recognized as a hallmark of malignancy in various different cancers [222,223]. In a phenomenon called the Warburg effect, cancer cells tend to undergo aerobic glycolysis where they rely on glycolysis instead of oxidative phosphorylation, even in the presence of oxygen [223]. This dysregulated increase in glucose influx and glycolytic rate is thought to provide energy on a large scale while depleting the TME of nutrients other cells needed by other cells. While this “Warburg” phenotype is tumor-dependent, TNBC has been shown to be more dependent on glycolysis compared to other breast cancer subtypes, as they overexpress glycolytic components such as lactate dehydrogenase (LDHA), glucose transporter 1 (GLUT1), and monocarboxylate transporters (MCT1/4) [224,225,226]. Some TNBC tumors overexpressed the GTPase-activating protein USP6NL which is involved in regulating signal transduction and upregulation of GLUT1 via the Wnt/β-catenin pathway [227]. The knockdown of USP6NL has been shown to inhibit TNBC cell growth, motility, and EMT [228]. TNBC cells are also known to be reliant on an increase in fatty acid oxidation and glutamine metabolism as an alternative energy source and to sustain the increased rate of cell growth [229,230].

### 6.2. Aerobic Glycolysis and Immunosuppression

Perhaps, one of the most intriguing advantages for tumor cell metabolic redirection is its influence on immune cell infiltration, where an immunosuppressive environment is created within the TME. The increased efflux of lactate that is typical in a glycolytic phenotype, for example, results in the acidification of the TME, which has been shown to inhibit CD8^+^ T-cell activity and TH_1_ cell IFN*γ* production [231,232], while the depletion of glucose due to the increased competitive uptake by cancer cells leads to cytotoxic immune cell dysfunction. In TNBC, LDHA expression was shown to increase the number of T_regs_ and reduce the infiltration of CD8^+^ T-cells [233]. In the same study, Haung et al. showed through a Kaplan–Meier survival analysis that co-expression of PD-L1 and LDHA in TNBC was linked to poor outcomes in patients with shorter OS and DFS [233]. Interestingly, they show that an over-expression of PD-L1 on TNBC cells results in an increase in LDHA and vice versa, identifying a therapeutic strategy to simultaneously inhibit metabolic and immunologic aspects of tumorigenesis by co-targeting LDHA and PD-L1 [233]. Feng et al. uncovered a role for TAZ, a YAP paralogous transcription cofactor and downstream effector of the Hippo pathway in the interplay between immunosuppression and aerobic glycolysis [234]. In their study, they show that a lactate-mediated increase in PD-L1 was dependent on TAZ in glycolytic cancer cells [234]. Furthermore, inhibiting the CSC-related Wnt pathway could aid in the decreased acidification of the TME and increased immune filtration [235]. The interplay of aerobic glycolysis and immunology in TNBC remains largely unexplored, with more players yet to be identified.

### 6.3. Glutamine Metabolism in Immunosuppression

Cancer cells also rely on glutamine metabolism for cell growth and anabolic processes. TNBC cells showed an increased reliance on glutamine uptake and metabolism where the glutamine transporters alanine, serine, cysteine-preferring transporter 2 (ASCT2), and L-type amino acid transporter 1 (LAT1) are over-expressed [236]. Once in the cell, glutamine is converted to glutamate and α-ketoglutarate, which could be converted to malate and then to pyruvate, effectively supplementing aerobic glycolysis and contributing to the Warburg phenotype. Lampa et al. reported that suppression of glutaminase synergized the inhibitory effect of mammalian target of rapamycin (mTOR) on the growth of TNBC cell lines [237]. However, a recent study by Leone et al. also used a novel glutaminase antagonist, JHU083, that inhibits glutamine-requiring enzymes [238]. They found that treatment with JHU083 reverted the Warburg effect and inhibited glycolysis, thus increasing the glucose and glutamine content in the tumor as well as increasing the infiltration of CD8^+^ T-cells [238]. Metabolic analysis showed that the glutamine antagonist increased oxidative phosphorylation through an upregulation of mitochondrial proteins in T-cells but suppressed overall metabolism in cancer cells. While targeting glutamine uptake seems to be a plausible therapeutic strategy, it is limited by the fact that glutamine uptake is also essential for immune cell function [239]. However, combining JHU083 with immunotherapy led to a great response in vivo, where the mice treated with the glutamine antagonist and anti-PD-1 generated significant antitumor effects, with complete response rates close to 100% [238]. While the safety of the glutamine antagonist has yet to be determined, this work provides a glimmer of hope for simultaneously inhibiting metabolic reprogramming and activating the anti-tumor response as a means of therapy.

### 6.4. Lipid Metabolism in Immunosuppression

TNBC cells exhibit an increase in fatty acid oxidation (FAO) and a decrease in fatty acid synthesis (FAS) compared to other subtypes [239]. Specifically, FAO seems to be crucial in the maintenance of breast CSCs. Studies have reported a higher FAO rate in TNBC CSCs than non-CSCs [240,241]. Wang et al. found that FAO in breast CSCs is dependent on STAT3 signaling, identifying a possible avenue to target lipid metabolic rewiring through the inhibition of JAK/STAT3 [241]. Their work also establishes a link between chemoresistance and FAO levels, where blocking FAO re-sensitized cells to chemotherapy in vivo [241]. This was consolidated by Casciano et al., who reported a link between the highly amplified MYC transcription factor (which occurred in up to 50% of TNBC cases) and its role in promoting FAO [242]. While the role of MYC in TNBC metabolism remains largely unknown, it may be considered as a potential therapeutic avenue in the future.

Lipid metabolism also plays a role in immune cell development and activation. T_reg_ cells adapt to the nutrient depleted hypoxic TME by metabolically depending on fatty acids. T_regs_ relies on FAO for energy to proliferate and exert an immunosuppressive function [243], while T-cell activation is dependent on FAS [244]. Furthermore, increased lipid uptake upregulated PD-1 in CD8^+^ T-cells, while PD-1 blockade activated these T-cells. Given that lipid metabolism is a crucial aspect of TNBC tumorigenicity and T_reg_ function, targeting enzymes involved with FAO could potentially lead to a reduction in TNBC tumor burden, CSC enrichment, and enhance anti-tumor immunity. While pharmacologically targeting FAO in TNBC has garnered preclinical success, more work is still required to decipher the effect on immune cell infiltration [245].

Emerging evidence points to cholesterol as another culprit in the interplay between immune evasion and metabolic reprogramming, as well as CSC enrichment. Cholesterol is a key component in the cell membrane and acts as an important signaling molecule essential to cell growth and survival [246]. Breast CSCs seem to rely heavily on cholesterol synthesis, possibly for the maintenance of the desired level of membrane fluidity. Reduced membrane cholesterol levels are associated with metastasis, whereas high membrane cholesterol levels and further changes in membrane biophysical properties are associated with increased chemoresistance in breast cancer cells [247]. Increased cholesterol synthesis was associated with shorter relapse-free survival, and a recent study showed that inhibition of cholesterol synthesis pathway reduced breast CSC enrichment [248]. Some reports have shown that the inhibition of cholesterol synthesis pathways using statins, or inhibition of its master regulator RAR-related orphan nuclear receptor γ (RORγ), induced TNBC tumor regression [249,250]. The cholesterol synthesis pathway also overlaps with CSC-related pathways, such as YAP signaling. The cholesterol-lowering drug Simvastatin is currently in clinical trials to treat breast cancer, which indirectly inhibits YAP through the inhibition of HMG-CoA-Reductase [251]. Proprotein convertase subtilisin/kexin 9 (PCSK9) monoclonal antibodies or vaccinations work to reduce cholesterol and have also been proposed to improve clinical outcomes in breast cancer patients [252]. Cholesterol metabolism also plays a role in immune cell activity, as they depend on their membrane to function [253]. A study by Ma et al. showed that cholesterol in the TME influences CD8^+^ T-cells, leading to the expression of immune checkpoint molecules, such as PD-1 [254]. High levels of cholesterol were associated with low anti-tumor immunity, which was restored upon reducing cholesterol [255]. The effect of drugs such as Simvastatin on immune cell infiltration remain open to investigation.

### 6.5. Autophagy in TNBC

One of the major players in the immunometabolic landscape of TNBC may be autophagy. Autophagy is a process in which intracellular constituents are degraded or recycled to regulate metabolic pathways under nutrient deprivation to maintain cell survival [256]. A marker of autophagy, microtubule-associated protein 1 light chanin 3B (LC3B) is highly expressed in TNBC and associated with poor clinical outcomes [257]. Glycolysis or GLUT1 inhibitors have been shown to induce autophagy deficiency and eventual cell death in TNBC cells [258,259]. Interestingly, the anti-CD73 antibody 1D7 was shown to mediate autophagy and inhibit the motility of TNBC cells [260]. Wen et al. further demonstrated that the inhibition of autophagy sensitized TNBC cells to chemotherapeutic agents [261]. Glutamine antagonsists have also been discussed as potential therapeutic targets; however, glutaminase inhibition accelerates autophagy and upreuglates FAO as a means for tumor cell survival [262]. Autophagy directly promotes FAO by providing the mitochondria with free fatty acids, leading to the accumulation of lipid droplets [263]. Furthermore, autophagy has been shown to hinder T-cell-mediated anti-tumor activity against TNBC both in vitro and in vivo [264]. As such, the interplay between metabolic pathways and autophagy in TNBC requires further investigation.

### 6.6. Interplay of HIF-1α in Cancer Metabolism and Immunosurveillance

In addition to the previously described importance of HIF-1α in TNBC angiogenesis, its role in metabolic reprogramming provides another therapeutic avenue in breast cancer cells and CSCs. HIF-1α activity in response to hypoxia leads to the expression of glycolytic enzymes and contributes to the Warburg effect, which increases the acidification of the TME and decreases immune cell infiltration [255,265]. Work by Bharti et al. used high-resolution ^1^H MRS (in vivo proton magnetic resonance spectroscopy) imaging in the aqueous and lipid phases of HIF-silenced tumors, after which the metabolic profiles were elucidated to determine the effect of HIF-1/2α inhibition [255]. They found that with HIF-1α silencing in TNBC, amino acids such as glutamine were decreased, along with lipid signals and droplets, suggesting that HIF-1α plays a role in TNBC metabolic adaptation [265]. Additionally, a study by Lee et al. discovered a strategy whereby silencing the oxidative stress master regulator NRF2 reduced HIF-1α accumulation and hindered HIF-1α induction of glycolysis-related genes [266]. In breast cancer cell lines, HIF-1α was found to increase the expression of adenosine receptor 2B (A2BR), which, as mentioned in earlier sections, plays a role in MDSC expansion and immunosuppression [267].

In breast cancer, HIF-1α also controls the expression of cluster of differentiation 47 (CD47), an integrin membrane protein expressed on many different cell types for the regulation of a wide range of cellular processes [268,269]. Specifically, cancer cells have been shown to overexpress CD47, where it forms a complex with signal-regulatory protein α (SRP- α) on phagocytes and inhibits macrophage-mediated phagocytosis of the TNBC cells [270]. CD47 expression is a well-known strategy by which tumor cells escape immunosurveillance. Researchers have explored blocking CD47 to induce a wide range of anti-tumor immune function [271,272,273,274]. High expression of CD47 in TNBC was associated with unfavorable prognosis, EMT signals, and metastasis [270]. Furthermore, a study by Kaur et al. showed that the blockade of CD47 was effective in TNBC CSC suppression and downregulation of stem-cell related pathways [274]. The preclinical success of targeting CD47 led to the therapy moving on to phase I clinical trials [275]. Interestingly, CD47 was recently shown to promote a Warburg phenotype by protecting the ubiquitin mediated degradation of ENO1, a glycolytic enzyme, providing another role of CD47 in cancer metabolic rewiring, in addition to its established role in immune evasion [276]. HIF-1α is also involved in decreasing the anti-tumor immune response via its control of the PD-1/PD-L1 in immune cells and tumor cells. The hypoxia-inducible element (HRE), where HIF-1α binds, was found in the PD-L1 proximal promoter [277]. Combined, the above information suggests that HIF-1α might serve as a therapeutic strategy to overcome both TNBC metabolic rewiring and immunosuppression.

## 7. Conclusions

Immune checkpoint inhibition has evolved significantly to reflect the immunogenic potential of TNBC, among other cancers. Despite some positive clinical outcomes with anti-PD-1/PD-L1 and anti-CTLA-4 antibodies, the monotherapy only provides benefit to a subset of the patients, warranting further studies. To date, a large number of clinical trials are looking at immune checkpoint inhibition as a treatment modality to complement chemotherapy. Since enrichment of CSCs and tumor reconstitution are often associated with chemotherapeutic agents, novel combination strategies include normalizing tumor-associated vasculature, modulating the TME, and targeting a multitude of receptors and transcription factors, which may lead to a more effective and durable response for TNBC treatment. In addition to active immunity, therapies that strengthen passive immunity or counter the metabolic reprogramming of tumors may be advantageous in future research. Although significant progress has been made, many challenges remain in the field when looking for a combinatorial immunotherapy to target TNBC.

## Figures and Tables

**Table 1:** List of completed or ongoing clinical trials using PD-1/PD-L1 immune checkpoint inhibitors.

Trial (National Clinical Trial Identifier)	Phase	Condition	Interventions	Key Results	Reference
**Atezolizumab** **Monotherapy (NCT01375842)**	I	Locally advanced or metastatic solid tumors	(1) Atezolizumab (2) Placebo	**Median PFS:** 4.0 months for arm (1); 1.8 months for arm (2) **Median OS:** 17.6 months for arm (1); 7.3 months for arm (2) **Incidence of trAEs:** 62% for arm (1); 43% for arm (2)	[23]
**IMpassion130** **(NCT02425891)**	III	Previously untreated metastatic TNBC	(1) Atezolizumab + Nab-Paclitaxel (2) Placebo + Nab-Paclitaxel	**Median PFS:** 7.2 months for arm (1); 5.5 months for arm (2) **Median OS:** 21.3 months for arm (1); 17.6 months for arm (2) **Incidence of grade 3+ trAEs:** 15.9% for arm (1); 8.2% for arm (2)	[24]
**KEYNOTE-086** **(NCT02447003)**	II	Metastatic TNBC	(1) Pembrolizumab	**Median PFS:** 2.0 months for previously treated; 2.1 months for PD-L1^+^ tumors **Median OS:** 9.0 months for previously treated; 18.0 months for PD-L1^+^ tumors **Incidence of trAEs:** 60.6% for previously treated; 63.1% for PD-L1^+^ tumors	[25,26]
**KEYNOTE-335** **(NCT02819518)**	III	Previously untreated, locally recurrent, inoperable or metastatic TNBC	(1) Pembrolizumab + Chemotherapy (2) Placebo + Chemotherapy	**Median PFS:** 9.7 months for arm (1); 5.6 months for arm (2) **ORR:** 53% for arm (1); 40% for arm (2) **Incidence of trAEs:** 68.1% for arm (1); 66.9% for arm (2)	[27]
**IMpower110** **(NCT02409342)**	III	Stage IV non-squamous or squamous non-small cell lung cancer	(1) Atezolizumab (2) (Carboplatin/Cisplatin) + (Pemetrexed/Gemcitabine)	**Median OS:** 17.5 months for arm (1); 14.1 months for arm (2) **Incidence of trAEs:** 60.5% (**grade 3 +:** 12.9%) for arm (1); 85.2% (**grade 3 +:** 44.1%) for arm (2)	[31]
**IMpassion131** **(NCT03125902)**	III	Previously untreated, locally advanced or metastatic TNBC	(1) Atezolizumab + Paclitaxel (2) Placebo + Paclitaxel	**Median PFS:** 6.0 months for arm (1); 5.7 months for arm (2) **Median OS:** 22.8 months for arm (1); 22.1 months for arm (2)	[32]
**KEYNOTE-119** **(NCT02555657)**	III	Metastatic TNBC	(1) Pembrolizumab (2) Chemotherapy	**Median PFS:** 2.1 months for arm (1); 3.3 months for arm (2) **Median OS:** 9.9 months for arm (1); 10.8 months for arm (2) **Incidence of grade 3+ trAEs:** 14% for arm (1); 36% for arm (2)	[34]

PFS: progression-free survival; OS: overall survival; ORR: objective response rate; trAEs: treatment-related adverse events.

**Table 2:** List of completed or ongoing clinical trials using immune checkpoint inhibitors in combinatorial therapy.

Trial (National Clinical Trial Identifier)	Phase	Condition	Interventions	Key Results	Reference
**IMbrave150 (NCT03434379)**	III	Locally advanced or metastatic solid tumors	(1) Atezolizumab + Bevacizumab (2) Sorafenib	**Median PFS:** 6.8 months for arm (1); 4.3 months for arm (2) **Median OS: 2 months:** 67.2% for arm (1); 54.6% for arm (2) **Incidence of grade 3+ trAEs:** 56.5% for arm (1); 55.1% for arm (2)	[81]
**(NCT02536469)**	I	Advanced malignant solid tumors	(1) HuMax-IL-8	No objective tumor responses observed, 73% had stable disease at week 24 Serum IL-8 significantly reduced on day 3, relative to baseline (*p* = 0.0004) **Incidence of trAEs:** 33% (mostly grade 1)	[85]
**MAGIC-8** **(NCT03689699)**	I/II	Hormone-sensitive prostate cancer	(1) Nivolumab (2) Nivolumab + BMS-986253	**No preliminary data available,** results expected in 2022	[86]
**(NCT02754141)**	I/II	Malignant solid tumors	(1) BMS-986179 (2) Nivolumab + BMS-986179	**Incidence of trAEs:** N/A for arm (1); 58% for arm (2) **Incidence of grade 3 trAEs:** N/A for arm (1); 15% for arm (2) Overall, both arms (1) and (2) are well-tolerated	[87]
**(NCT01302405)**	I	Advanced solid tumors	(1) PRI-724	**Incidence of trAEs:** 17% **Incidence of grade 3+ trAEs:** 11.1%PRI-724 has an acceptable toxicity profile	[88]
**SYNERGY** **(NCT03616886)**	I/II	Previously untreated, locally recurrent, inoperable or metastatic TNBC	(1) Paclitaxel + Carboplatin + Durvalumab + Oleclumab	**No preliminary data available,** results expected in 2023	[89]

PFS: progression-free survival; OS: overall survival; trAEs: treatment-related adverse events.
